# Supplementary material for: A Randomized Controlled Trial of a Parent-Led Memory-Reframing Intervention to Reduce Distress and Pain Associated with Vaccine Injections in Young Children
Source: Children (Basel). 2023 Jun 22;10(7):1099. doi: 10.3390/children10071099 (PMC10378095; doi:10.3390/children10071099)
Supplement: Supplementary file 1 [file children-10-01099-s001.zip › children-2444624-supplementary.pdf]

Figure S1: Evidence-based pain management strategies

skip solutions for kids in pain
pour la douleur chez les enfants

Needle Pain & Anxiety Management for Vaccinations

Resources for Children, Parents, and Families

Parents Canada 1-Page Resource

Access here: tinyurl.com/ddebsvdy

Meg Foundation

Access here: megfoundationforpain.org

Conversation Canada Articles

5 simple ways to ease vaccination pain for yourself and your child: tinyurl.com/ccmhzcp2

Needle fears can cause COVID-19 vaccine hesitancy, but these strategies can manage pain and fear: tinyurl.com/23xfpay6

Government of Canada Resources

What to expect at your child's vaccination appointment: tinyurl.com/5enbyvnc

À quoi s'attendre lors de la visite de vaccination: tinyurl.com/e49yjzbu

CARD System: Comfort, Ask, Relax, Distract

Access here: tinyurl.com/2cc82n7j

Mom Hack Video: When Kids are Afraid of Needles

Access here: tinyurl.com/445k97fe

It Doesn't Have to Hurt Video

Watch here: tinyurl.com/2esx7k26

For more #ItDoesntHavetoHurt content: www.itdoesnthavetohurt.ca

Anxiety Canada

Helping Children with Needle/Vaccine-Related Anxieties: tinyurl.com/uwatncxk

My Anxiety Pain Plan
- Children & teens: tinyurl.com/n6em75kw
- Adults: tinyurl.com/yv8pjbb8

Les stratégies Tout doux

Téléchargez ici: tinyurl.com/hr3aczpw

Commitment to Comfort

Access here: tinyurl.com/vancepcb

J'aime pas les piqûres !

Téléchargez ici: tinyurl.com/ra8syhmk

Be Sweet to Babies Video Series Available in several languages

Watch here: tinyurl.com/ur2e9vf9

Resources for Health Professionals and Adults

Immunize Canada Resources

For clinicians: tinyurl.com/279vhnvf
- Children: tinyurl.com/yacva6bd
- Youth: tinyurl.com/362zx5bc
- Adults: tinyurl.com/26reyw2a

La peur des aiguilles et ses répercussions: tinyurl.com/38c59wky

Clinical Practice Guidelines

For needle pain: tinyurl.com/495jnhk3
For needle fear: tinyurl.com/36zksk2b

Executive Summary/Sommaire Exécutif

COVID-19 Vaccine Hesitancy and Needle Fear Survey: tinyurl.com/3fja3yxj
Enquête sur la réticence à la vaccination contre la COVID-19 et la peur des injections: tinyurl.com/z6eerzau

kidsinpain.ca #ItDoesntHaveToHurt @kidsinpain

Figure S2: Memory-reframing intervention handout

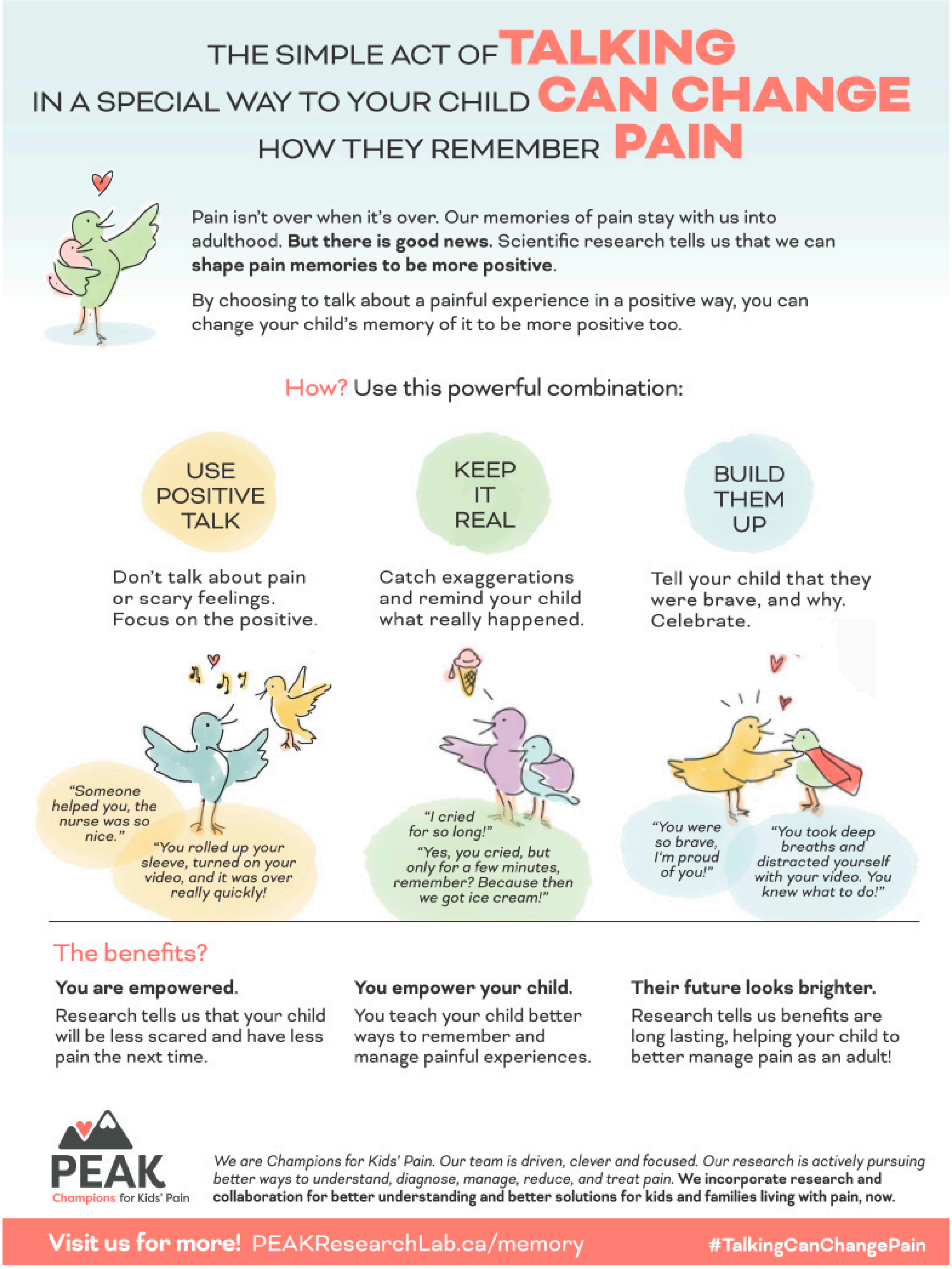

Table S1: Parent Adherence Checklist

Question	Answer Options
1. Before or during the first COVID shot, which of these strategies did you use (check all that apply)	• Created a Comfort Plan for my child<br>• Talked about the upcoming COVID shot<br>• Used numbing creams and patches<br>• Practiced relaxation techniques (e.g., deep breathing)<br>• Used neutral language rather than drawing attention to pain<br>• Avoided reassuring my child (e.g., don't say "It will be over soon" or "You are fine")<br>• Distracted my child (e.g., with a video, music)
2. After the first COVID shot, which of these strategies did you use (check all that apply)	• Talked about positive things that happened before/during /after the shot<br>• Tried to avoid using pain language (e.g., saying hurt, pain, stab)<br>• Caught any exaggerations regarding needle pain and/or fear and reminded my child what really happened<br>• Told my child they were brave<br>• Talked about what helped to deal with needle pain and/or fear
3. Before or during the second COVID shot, which of these strategies did you use (check all that apply)	• Created a Comfort Plan for my child<br>• Talked about the upcoming COVID shot<br>• Used numbing creams and patches<br>• Practiced relaxation techniques (e.g., deep breathing)<br>• Used neutral language rather than drawing attention to pain<br>• Avoided reassuring my child (e.g., don't say "It will be over soon" or "You are fine")<br>• Distracted my child (e.g., with a video, music)
4. After the second COVID shot, which of these strategies did you use (check all that apply)	• Talked about positive things that happened before/during /after the shot<br>• Tried to avoid using pain language (e.g., saying hurt, pain, stab)<br>• Caught any exaggerations regarding needle pain and/or fear and reminded my child what really happened<br>• Told my child they were brave<br>• Talked about what helped to deal with needle pain and/or fear
